# Monitoring of Ozone Risk for Forests in the Czech Republic: Preliminary Results

**DOI:** 10.1100/tsw.2007.84

**Published:** 2007-03-21

**Authors:** Vít Šrámek, Radek Novotný, Emilie Bednářová, Hana Uhlířová

**Affiliations:** ^1^ Forestry and Game Management Research Institute Jíloviště-Strnady, 156 04 Praha5, CZ, Czech Republic; ^2^ Mendel University of Agriculture and Forestry, Zemědělská 3, 613 00 Brno, CZ, Czech Republic

**Keywords:** ozone, forest health, defoliation, epicuticular waxes, malondialdehyde

## Abstract

Ozone (O_3_) is supposed to represent a significant risk for the health of forest ecosystems in Central Europe. So far, however, its impact on stands growing under natural conditions has not been clearly proved. A new project of the National Agency for the Research in Agriculture is focused on the O_3_ effect on selected parameters of forest health. This paper presents the results of the first year of monitoring, 2005. In 2005, high O_3_ concentrations were measured, mainly in the spring. In the summer, due to wet and cold weather, the O_3_ load was comparatively low. In the plots investigated, the concentrations of O_3_ were higher with the altitude. The amount of epicuticular waxes on 1-year-old Norway spruce needles was the only factor showing significant correlation to O_3_ concentration. Defoliation of the stands depended only on the stand age. The amount of malondialdehyde (MDA), an oxidative stress marker, was related to the altitude, and only for European beech. The results are preliminary, as the summer O_3_ development was not typical in 2005, and the results may change over the next monitoring periods.

## INTRODUCTION

Central Europe is well known for the vast forest damage due to high sulfur dioxide ($SO_2$) concentrations in the 1970s and 1980s[1]. Since 1989, $SO_2$ concentrations were significantly decreased. From 1989–2005, the limit value for the health protection of the ecosystems and vegetation of 20 $\mu g.m^{-3}$ was exceeded, on average, at less than 1% of the region of the Czech Republic[2]. Under such conditions, other factors negatively affecting the health of the forest ecosystems start to be of interest. From the air pollution viewpoint, besides acid deposition, it is mainly the impact of ozone ($O_3$)[3,4]. Ozone stress in Central Europe, especially regarding the $O_3$ flux, is considered to be of importance[5,6] and even of possible negative effect on the economy of forest management[7]. Other studies, however, indicate that $O_3$ may not be more important than the other natural stress factors[8].

To consider the impact of $O_3$ on the forest stands in the Czech Republic, a monitoring project was proposed for the 2005–2008 period, based on combined evaluation of the health of forest tree species, $O_3$ concentrations, selected markers of the impact of this harmful agent, and on visual assessment in natural conditions. The aim of the project was to reveal whether the real response of spruce and beech stands in conditions of the Czech Republic to today's $O_3$ concentrations can be proved. To characterize the state of health, the following parameters for long-term investigation have been selected: defoliation[9], amount of epicuticular waxes[10], and amount of malondialehyde (MDA), known as a product of peroxidation of the cell-membrane lipids[11,12]. This paper presents the preliminary results of the project in 2005.

## METHODS

The project is based on eight plots of Intensive Monitoring of the Forest Ecosystems (ICP Forests, Level II), selected for their individual sensitivity to $O_3$ damages. Nearby, complementary plots to each of these plots were installed to cover the three age stages of the two main tree species, Norway spruce (*Picea abies,* [L.] Karst) and European beech (*Fagus sylvatica*, L.) in individual regions. In 2005, a total of 48 plots were assessed. The age of the stand was characterized by the age class (10-year interval) based on forest management inventory.

Defoliation was assessed in all plots of 25 trees using the method determined by the ICP Forests Programme[13]. During August and September 2005 (beech) and September to October 2005 (spruce), samples of assimilation organs of the sun-exposed part of the crown were taken from five trees at each of the plots. Mixed samples from each plot were analyzed for MDA amounts[14], indicating oxidative stress. At the eight "parent" spruce and beech plots, analysis of the structure and amount of epicuticular waxes was done[15]. For spruce, both the current-year and 1-year-old needles were analyzed.

Ozone concentrations during the vegetation period were measured using the passive $O_3$ samplers Gradko (Gradko International Ltd., GB; accuracy ±20%; detection limit 2 $\mu g.m^{-3}$). Exposition was done in a 4-week run; analysis of the samplers was done by the producer. Ozone monitoring covered the eight "Level II" monitoring plots only. Because the complementary plots are mostly situated at the same altitudes and within a short distance (<3 km), the data were also related to these plots for the purpose of statistical evaluation.

Other parameters (meteorological factors, soil water potential, visual assessment of damage to ground vegetation) are not included in this paper. For statistical evaluation, correlation and cluster analyses were done using the Statistica and QCExpert software.

## RESULTS AND DISCUSSION

The development of $O_3$ concentration was not typical in 2005 (Fig. 1). The highest concentrations were measured just at the beginning of the vegetation period. Summer values were relatively low due to cold and wet weather in July. All the stations have shown similar development of $O_3$ during the vegetation season. As expected, the top concentrations were measured within the highest altitude plot Švýcárna (1300 m a.s.l.), and the lowest in the plot Želivka (558 m a.s.l.). The differences among the stations were comparatively stable; in individual measuring periods, the variation ranged mostly from 40–50 $\mu g.m^{-3}$.

The average defoliation of the Norway spruce stands was 22.3% in 2005. In individual plots, it oscillated from 9–36%. Defoliation over 30% was assessed at five spruce stands older than 50 years. For European beech, the average defoliation was slightly lower (18.6%), ranging from 8.7–31%. Defoliation higher than 30% was found only in the two beech stands over 130 years old.

MDA amounts were highest in the 1-year-old needles of Norway spruce, where the average value was 7.44 $\mu mol.g^{-1}$. In the current-year needles (6.15 $\mu mol.g^{-1}$), the MDA amount was only slightly higher than in the beech leaves (6.03 $\mu mol.g^{-1}$), which can be explained by the short time of exposure to oxidative stress.

A higher amount of epicuticular waxes, as expected, was in the European beech leaves, ranging from 7.3–8.6% of the leaf dry mass. Quantitative differences among localities were well corresponding to disturbances of the structure of wax layer (Figs. 2 and 3). For Norway spruce, the wax amounts were lower. In the current-year needles, they ranged from 1.4–2.0% of the needle dry mass; in the 1-year-old needles, the range was from 1.1–1.9%. In both species, the correlation of epicuticular wax degradation and the altitude was obvious.

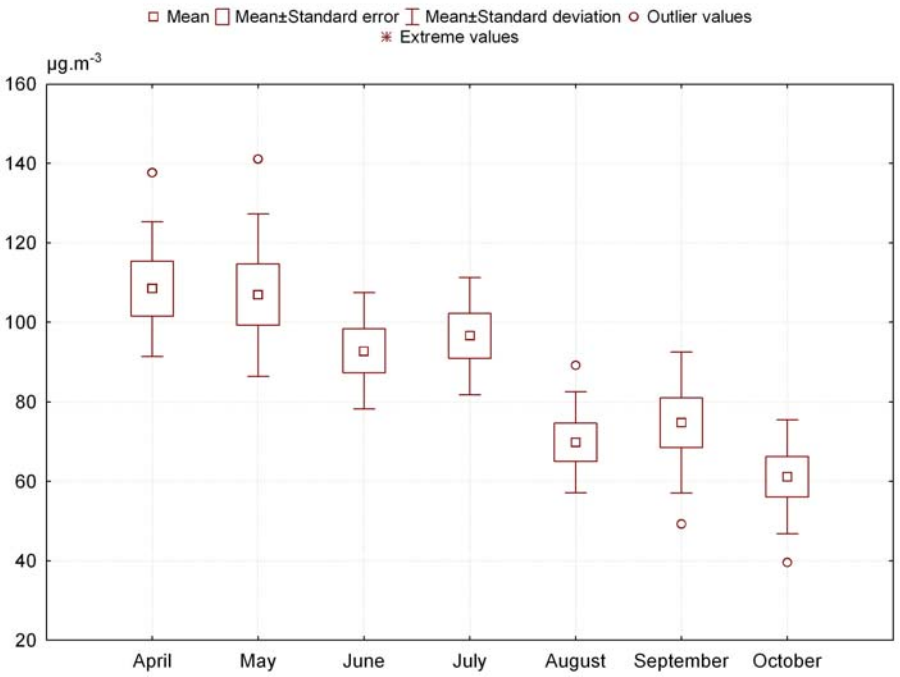

**FIGURE 1**. Development of $O_3$ monthly mean concentrations at monitored localities.

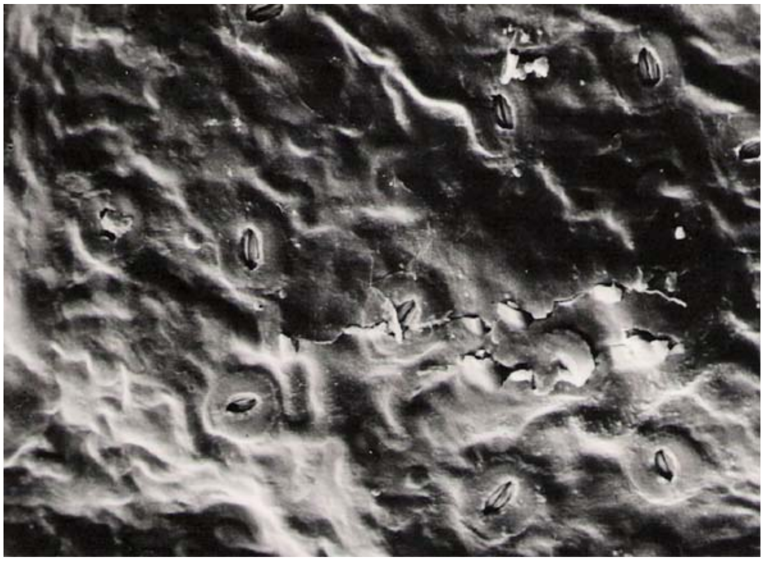

**FIGURE 2.** Degraded structure of epicuticular waxes, European beech, Vidly, 950 m a.s.l.

The matrix of correlation of individual parameters for European beech (Table 1) shows that, in the beech stands, there is a significant correlation of tree defoliation and stand age. Also, the amount of MDA and epicuticular waxes depends on the altitude. For spruce, there is also a significant correlation (Table 2) of defoliation and stand age, and of epicuticular waxes and the altitude. Even significantly growing $O_3$ concentration with the altitude is clearly visible. The relation of MDA and epicuticular waxes in between the current year and 1-year-old needles is quite clear. The results of partial correlation confirmed a significant relation in the same cases.

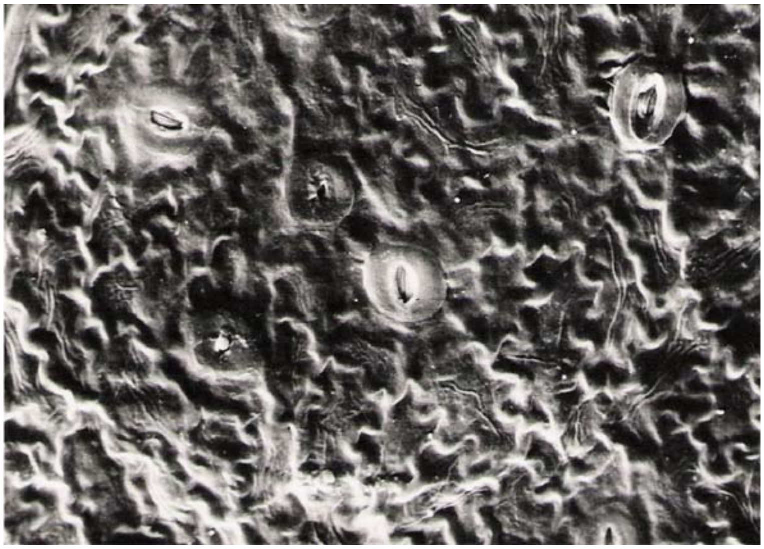

**FIGURE 3.** Undamaged structure of epicuticular waxes, European beech, Buchlovice, 350 m a.s.l.

**TABLE 1**
**Matrix of Correlation for European Beech**

| | Altitude | Age Class | MDA | $O_3$ | Defoliation |
|---|---|---|---|---|---|
| **Age class** | 0.2274 | | | | |
| | $p$ = 0.309 | | | | |
| **MDA** | **0.6438** | 0.3390 | | | |
| | ***p* = 0.001** | $p$ = 0.123 | | | |
| **$O_3$** | 0.4047 | –0.0185 | 0.3682 | | |
| | $p$ = 0.062 | $p$ = 0.935 | $p$ = 0.092 | | |
| **Defoliation** | 0.2507 | **0.7406** | 0.4025 | –0.2149 | |
| | $p$ = 0.260 | ***p* = 0.000** | $p$ = 0.063 | $p$ = 0.337 | |
| **Waxes** | **–0.9627** | –0.3696 | **–0.9519** | –0.4730 | 0.2497 |
| | ***p* = 0.037** | $p$ = 0.630 | ***p* = 0.048** | $p$ = 0.527 | $p$ = 0.750 |

The relation of tree defoliation and stand age has already been proved by many authors[16,17,18]. Surprisingly, the correlation of defoliation and altitude was not significant, although altitude is often considered to be a factor influencing tree health[18,19]. This can be explained by the fact that monitoring was focused on the age gradients in the nearby localities, thus the impact of the altitude has been overlapped. Monitoring that focused on the $O_3$ impact in different altitudes was done in 2006.

The amount of epicuticular waxes shows correlation to $O_3$ concentrations in 1-year-old Norway spruce needles. This could be connected to the wax degradation[10] or to disturbances in its production[20] due to $O_3$, which are probably pronounced after a longer period (more than one vegetation season) of exposure. However, intercorrelation to the altitude has to be considered. Connected changes in meteorological factors can be more important with respect to the wax layer forming than $O_3$ itself.

**TABLE 2**
**Matrix of Correlation for Norway Spruce**

| | Altitude | Age Class | MDA* | MDA** | $O_3$ | Defoliation | Waxes* |
|---|---|---|---|---|---|---|---|
| **Age class** | –0.0061 | | | | | | |
| | *p* = 0.978 | | | | | | |
| **MDA*** | 0.2036 | –0.0809 | | | | | |
| | *p* = 0.340 | *p* = 0.707 | | | | | |
| **MDA**** | 0.1913 | –0.2183 | **0.7611** | | | | |
| | *p* = 0.370 | *p* = 0.305 | ***p* = 0.000** | | | | |
| **$O_3$** | **0.7015** | –0.0638 | 0.1605 | 0.0530 | | | |
| | ***p* = 0.000** | *p* = 0.767 | *p* = 0.454 | *p* = 0.806 | | | |
| **Defoliation** | 0.0238 | **0.7607** | 0.0092 | –0.1621 | –0.1263 | | |
| | *p* = 0.912 | ***p* = 0.000** | *p* = 0.966 | *p* = 0.449 | *p* = 0.557 | | |
| **Waxes*** | **–0.7816** | 0.2045 | –0.3493 | –0.5458 | –0.3871 | –0.3789 | |
| | ***p* = 0.022** | *p* = 0.627 | *p* = 0.396 | *p* = 0.162 | *p* = 0.343 | *p* = 0.355 | |
| **Waxes**** | **–0.9425** | 0.4485 | –0.3965 | –0.3695 | **–0.7501** | –0.5542 | **0.8165** |
| | ***p* = 0.000** | *p* = 0.265 | *p* = 0.331 | *p* = 0.368 | ***p* = 0.032** | *p* = 0.154 | ***p* = 0.013** |

* Current-year needles.

** 1-year-old needles.

Concerning environmental factors, MDA amounts show significant correlation only to the altitude, and only for European beech. Relatively low $O_3$ concentrations in the summer of 2005 can be one of the possible explanations. The oxidative stress was thus driven by other factors. For beech, this type of damage was growing with the altitude, probably also due to increasing intensity of meteorological stress and intensity of UVB radiation. For Norway spruce, oxidative stress could be increased also in lower altitudes, where trees have been affected by a dry period at the turn of August and September[21]. It can be supposed that when comparing longer periods of different $O_3$ development, the relations among individual parameters may become clearer.

## CONCLUSIONS

The evaluation of defoliation, MDA amount, and amount of epicuticular waxes and their relation to $O_3$ concentrations, stand age, and altitude in which they grow, has revealed some interesting facts in 2005. Defoliation was significantly correlated only to the stand age. The amount of epicuticular waxes was significantly growing with higher $O_3$ concentration in 1-year-old Norway spruce needles. These relations, however, could be partly affected by the intercorrelation of the two parameters to the altitude. MDA amounts have shown significant increase with the altitude, but only for European beech. The presented results should be considered as preliminary. They could be influenced by relatively low $O_3$ concentrations in the summer of 2005. Other, more precise, relationships can be expected for 2006, as the $O_3$ load in Central Europe that year was generally higher.

## ACKNOWLEDGMENTS

The work is supported by the National Agency for Agriculture Research (1G57045) and the Ministry of Agriculture of the Czech Republic (project 0002070201).
